# The genomic landscape of metastatic breast cancer: Insights from 11,000 tumors

**DOI:** 10.1371/journal.pone.0231999

**Published:** 2020-05-06

**Authors:** Jacob Rinaldi, Ethan S. Sokol, Ryan J. Hartmaier, Sally E. Trabucco, Garrett M. Frampton, Michael E. Goldberg, Lee A. Albacker, Anneleen Daemen, Gerard Manning

**Affiliations:** 1 Department of Bioinformatics & Computational Biology, Genentech Inc., South San Francisco, CA, United States of America; 2 Foundation Medicine, Cambridge, MA, United States of America; Mayo Clinic Rochester, UNITED STATES

## Abstract

**Background:**

Metastatic breast cancer is the leading cause of cancer death in women, but the genomics of metastasis in breast cancer are poorly studied.

**Methods:**

We explored a set of 11,616 breast tumors, including 5,034 metastases, which had undergone targeted sequencing during standard clinical care.

**Results:**

Besides the known hotspot mutations in *ESR1*, we observed a metastatic enrichment of previously unreported, lower-prevalence mutations in the ligand-binding domain, implying that these mutations may also be functional. Furthermore, individual *ESR1* hotspots are significantly enriched in specific metastatic tissues and histologies, suggesting functional differences between these mutations. Other alterations enriched across all metastases include loss of function of the CDK4 regulator *CDKN1B*, and mutations in the transcription factor *CTCF*. Mutations enriched at specific metastatic sites generally reflect biology of the target tissue and may be adaptations to growth in the local environment. These include *PTEN* and *ASXL1* alterations in brain metastases and *NOTCH1 alterations* in skin. We observed an enrichment of *KRAS*, *KEAP1*, *STK11* and *EGFR* mutations in lung metastases. However, the patterns of other mutations in these tumors indicate that these are misdiagnosed lung primaries rather than breast metastases.

**Conclusions:**

An order-of-magnitude increase in samples relative to previous studies allowed us to detect novel genomic characteristics of metastatic cancer and to expand and clarify previous findings.

## Background

Breast cancer is the most commonly diagnosed malignancy, and the leading cause of cancer death in women [1]. Virtually all breast cancer deaths are due to metastatic disease [2]. The process via which cancer cells disseminate from the primary tumor, colonize distal sites, and adapt to novel tumor microenvironments has not been fully characterized, but recent work implicates a cascade of genetic and epigenetic events that drive active degradation of the extracellular matrix, induce angiogenesis, enhance motility, promote immune evasion, and co-opt the epithelial-mesenchymal transition [3,4]. There is no cure for metastatic breast cancer and median survival is 18 to 24 months, representing an enormous unmet medical need [5]. Success in developing better treatments for breast cancer will primarily be determined by our ability to impede the metastatic process and treat metastatic disease, which is largely unchecked by current therapies [6,7]. A key component of this effort will be understanding how specific oncogenic events in individual patients lead to metastasis.

Breast cancer is the archetype for the use of molecular profiling to guide treatment decisions and develop targeted therapies [8]. The observation that a subset of breast cancers express the estrogen receptor (ER) led to the development of aromatase inhibitors, selective estrogen receptor degraders (SERDs) and selective estrogen receptor modulators (SERMs), which now make up a core component of standard clinical care for patients with ER positive disease [9–11]. Similarly, the discovery of epidermal growth factor receptor 2 (HER2/ERBB2) overexpression on the surface of breast cancer cells led to the development of the monoclonal antibodies trastuzumab and pertuzumab, now part of standard care for HER2 positive patients [12,13]. Because cancer is a genomic disease, genomic profiling holds the promise of extending the results of molecular profiling by further refining personalized treatment decisions and catalyzing the development of additional targeted therapies.

Most large-scale sequencing efforts in breast cancer have focused on primary tumors, where thousands of cancer genomes have been analyzed across multiple studies [14–17]. The most frequently reported alterations include mutations in *TP53*, *PIK3CA*, *GATA3*, *MAP3K1*, *AKT1*, and *CBFB*

; amplification of *HER2*, *MYC*, *FGFR1* and *FGF3/4*; deletion of *PTEN*, *RB1* and *CDKN2A/B*; and oncogenic germline polymorphisms in *BRCA1/2* [14,17]. This mountain of genomic information has led to targeted therapies, improved prognostic and predictive models, and innovative clinical trials that stratify patients by genomic phenotype. Clinical trials are ongoing for drugs that target patients with *BRCA1/2* mutations, *AKT1* mutations, *PIK3CA* mutations, and *FGFR* amplification, illustrating the utility of genomics to define patient populations and guide drug discovery [18–23].

Significantly less work has been done to characterize genomic alterations in metastatic disease because of the difficulty in gaining access to samples and the efficacy of adjuvant therapies. Initial efforts uncovered mutations in the ligand-binding domain of the estrogen receptor (*ESR1*) in 10–30% of metastatic breast cancer patients, an alteration that is largely absent in primary disease [24–27]. Recent work in small cohorts of ~100–1000 metastatic tumors suggests that the majority of alterations are shared between primary tumors and metastases, and that JAK/STAT and SWI/SNF pathways are dysregulated at higher rates in metastatic disease; these studies have also implicated genes involved in DNA damage repair, the MAPK pathway, and epigenetic regulators [28–32].

Here we demonstrate the utility of large-scale sequencing of metastatic breast cancer, in an unprecedented cohort of 4,512 local and 5,034 metastatic breast tumors (with an additional 1,357 lymph node biopsies and 713 tumors with ambiguous metastatic status) collected during standard clinical care. We hypothesized that relative to local disease, the genomic fingerprints of metastatic tumors are enriched for (i) mechanisms of acquired resistance (due to treatment history) and (ii) alterations that induce or accelerate metastasis. We find evidence for both classes of alterations, complementing recent metastatic profiling efforts in small cohorts with clinical annotation and/or matched primary and metastatic samples.

## Methods

### Tumor samples and sequencing

Samples were submitted to a CLIA-certified, New York State-accredited, and CAP-accredited laboratory (Foundation Medicine, Cambridge, MA) for next-generation sequencing (NGS)-based genomic profiling. The pathologic diagnosis of each case was confirmed by review of hematoxylin and eosin (H&E) stained slides, and all samples that advanced to nucleic acid extraction contained a minimum of 20% tumor cells. The samples used in this study were not selected and represent “all comers” to Foundation Medicine genomic profiling. Samples were processed in the protocol defined by solid tumors and hematological cancers as previously described [33,34]. A brief description is provided below.

For solid tumors, DNA was extracted from formalin-fixed, paraffin-embedded (FFPE) 10 micron sections. Adaptor-ligated DNA underwent hybrid capture for all coding exons of 287 or 395 cancer-related genes plus select introns from 19 or 31 genes frequently rearranged in cancer. Genes are listed in **S8 Table.**

Captured libraries were sequenced to a median exon coverage depth of >500x using Illumina sequencing, and resultant sequences were analyzed for base substitutions, small insertions and deletions (indels), copy number alterations (focal amplifications and homozygous deletions) and gene fusions/rearrangements, as previously described [33,34]. Frequent germline variants from the 1000 Genomes Project (dbSNP142) were removed. To maximize mutation-detection accuracy (sensitivity and specificity) in impure clinical specimens, the test was previously optimized and validated to detect base substitutions at a ≥5% mutant allele frequency (MAF), indels with a ≥10% MAF with ≥99% accuracy, and fusions occurring within baited introns/exons with >99% sensitivity [33]. Known confirmed somatic alterations deposited in the Catalog of Somatic Mutations in Cancer (COSMIC v62) are called at allele frequencies ≥1% [35].

### Statistical analyses

Enrichment analyses were conducted by performing a logistic regression to predict the variable of interest (for instance, local or met status) and then a Wald test on the coefficient of interest. In all cases, predicted probability of ER-positivity, HER2 status, and mutation load per megabase were used as additional covariates. Statistical analysis of count tables (ESR1 mutations by site, subtype by site) was performed using Fisher’s exact tests. Sets of probabilities (output of machine learning algorithms) were compared using Kolmogorov-Smirnov tests. To control for additional covariates when comparing outputs of the skin classifier a beta regression was performed with a Wald test on the coefficient of interest. Corrected p-values for enrichment analyses were calculated by permuting the variable of interest 1000 times and selecting the most significant genomic alteration. P-values from the true dataset were then compared with these 1000 iterations to estimate the probability that any p-value across all genomic alterations would be deemed as significant by chance. Alteration rates in local disease and metastases were compared using Mann-Whitney tests.

### Machine learning algorithms

All classifiers used the random forest algorithm [36], with an input feature set consisting of the mutation (short variant), copy number, and structural rearrangement status of each gene on the panel–excluding enriched genes for the tissue of origin classifiers, as well as the per-sample mutation load per megabase. In each case, not all features impact prediction–the training process selects a subset of features that are useful for the classification task. The random forest classifier was implemented using R 3.1.0 with the randomForest 4.6–7 and caret 6.0–30 packages [37–39]. Random forests are ensembles of decision trees, where each tree is fit on a random sample with replacement of the training set and each candidate split is made on a random subset of the features. This produces models that generalize well to unseen data. Ensemble methods work by combining many weak models to produce a prediction that is more accurate than the prediction made by a single strong model. Each tree has been trained on a different subset of the training samples and a different subset of features, making them relatively independent and representative of different hypotheses about how features map to class labels. By combining these different hypotheses, which will be accurate on different unseen samples, we can generate a combined prediction that is much less susceptible to over-fitting noise in the training set. To make a prediction on an unseen sample, the mode of the predictions of all the trees in the ensemble is used–in other words, every tree votes and the majority wins. The percentage of votes given to one class is the probability that the model assigns to a given prediction. Mutation calls were summarized such that any gene harboring at least one mutation (regardless of functional impact) was considered “mutated” for that gene. The *mtry* parameter, which determines the number of variables available for sampling at each tree node, was set using 10-fold cross-validation—and a new model with the optimal *mtry* value was trained after this cross-validation phase to avoid over-fitting. The random forest algorithms consisted of 5,000 trees, each fit with a stratified resampling of the data to rebalance classes.

For the molecular and histological subtype predictors, all available samples were used for training (ER status was generated with an algorithm to infer status from pathology reports with 95% accuracy, see Pathology Report Parser below)– 1405 samples for molecular subtype and 3959 for histological subtype. Five hundred samples in each class were used for indication prediction. All reported accuracies are on held-out test data. We assessed the importance of each feature to model performance by independently permuting each feature and assessing the resultant decrease in out-of-bag accuracy.

### SGZ (Somatic-Germline-Zygosity) determination

Somatic vs. germline origin and homozygous vs. heterozygous or sub-clonal state of variants identified was determined without a matched normal control as described previously [40]. Briefly, for each patient we generate a segmented genome-wide copy number model and calculate the minor allele frequency (MAF) based on the patient SNP profiles. We then model the copy number of the MAF taking the observed noise into account. Goodness of fit was assessed with a Gibbs sampling-based Markov chain Monte Carlo algorithm and a grid-sampling approach.

Somatic/germline/ambiguous prediction was calculated using a 2-tailed binomial test and α cutoff of 0.01. Mutations were called homozygous if all copies in the tumor carried the mutant allele, heterozygous if both the reference and the mutant alleles were present, or sub-clonal somatic if the somatic allele frequency was significantly lower than the expected allele frequency.

### Pathology report parser

We parsed the hormone status from patients’ pathology reports using optical character recognition (OCR) software and a set of scripts employing natural language processing techniques. We received electronic scan images of pathology reports stored as PDFs, and extracted and stored the text from the images using ABBYY Fine Reader Engine 11. To parse the hormone status from the text, we built a set of python scripts (Python 2.7) that used regular expressions and filtering to find the most likely hormone receptor status. Within several lines of the string “ER” or “Estrogen”, we searched for the closest string representing a possible status. Strings associated with ER-positive staining included “positive”, “detect”, and “expression”. Strings associated with negative staining included “negative” and “rare”. We also detected negation and switched the status accordingly (e.g., “ER staining was detected” vs. “ER staining was not detected”). We filtered for text commonly found in pathology reports associated with incorrect matches, such as text explaining the interpretation of stain statuses or bibliographical entries. Finally, we set a threshold for the maximum distance in number of characters between the strings “ER” or “Estrogen” and the status after finding that distance negatively correlated with accuracy in our training dataset (130 pathology reports from the breast carcinoma cohort from TCGA and 25 from FMI, both redacted of protected health information).

We validated and calculated the accuracy of our parsing strategy by testing on a set of 100 new, internal pathology reports. A trained pathologist hand-analyzed each report and provided us with a “gold-standard” dataset. By comparing the ER status parsed from the report to the gold-standard, hand-analyzed status, we found that our parsing strategy was 94% accurate.

### Visualization

All visualizations were created using ggplot2 2.2.1 in R 3.1.0, except for the lollipop visualization, which was created using Lollipops, and the cell diagrams, which were made using ComplexHeatmap 1.14 in R 3.1.0 [37,41–43].

### Ethics approval and consent to participate

Approval for this study, including a waiver of informed consent and a HIPAA waiver of authorization, was obtained from the Western Institutional Review Board (Protocol NO. 20152817)

## Results

### Overview of clinical data

**Fig 1:**
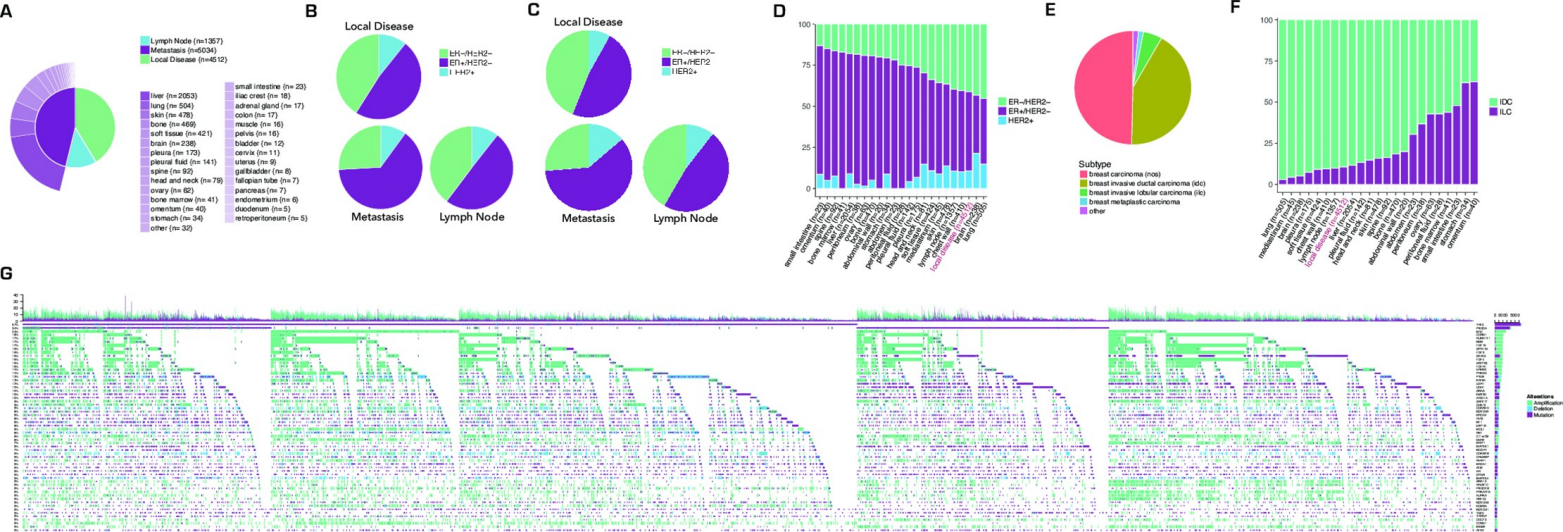
Overview of clinical and genomic data. (a) Frequency of biopsy sites for 10,903 sequenced breast cancer samples. An additional 713 tumors had an ambiguous classification. (b) Prevalence of *HER2* amplification and inferred ER status by biopsy site in 10,903 samples. ER status was inferred using a machine learning algorithm trained on 1,405 samples for which subtype was known–out-of-bag predictions are plotted for samples in training set, true values for these samples are shown in C (see **S1 Fig**). (c) Prevalence of *HER2* amplification and ER IHC status by biopsy site in 1,405 samples with complete clinical annotation. (d) Prevalence of inferred molecular subtype by metastatic site (n = 11293, note that some samples have a tissue biopsy site that confers ambiguous primary/met/ln status and 98 samples were from unknown sites). (e) Prevalence of histological subtype for all sequenced samples (n = 11616). (f) Prevalence of histological subtype by biopsy site Histological subtype was inferred using a machine learning algorithm trained on annotated data (see **S1 Fig**, n = 11293). (g) Landscape of genomic alterations in the cohort. Each cell represents the status of one gene in one patient, colored by alteration type (mutation, amplification, or deletion). Genes (rows) are sorted by alteration rate. Barplot shows alterations per sample, colored by type.

Samples from 11,616 breast cancer patients were submitted for targeted sequencing as part of standard clinical care. Thirty-nine percent were biopsied from local breast tumors (primary tumors or local recurrences), and 12% from lymph node metastases (**Fig 1A**). Notably, 5,034 (43%) were from distal metastases, comprising the largest collection of genomic profiles from metastatic breast cancer assembled to date. The median age was 55, with 1,343 patients under 40. Pathology reports containing ER status were available for 1,405 (12%) samples and were annotated with ER status using an automated algorithm. In the remaining cases ER status was imputed with an accuracy of 76% from mutation and copy number data using a machine learning algorithm trained on the samples with known ER status (**S1 Fig**). HER2 positivity was defined as *HER2* amplification as measured by the sequencing assay. Fifty-five percent of samples were scored as ER+/HER2-, with a significantly higher prevalence in metastatic samples (64% in metastases vs. 48% in local disease, p< 2.2e-16; **Fig 1B**), similar to the pattern seen in samples with clinical annotation (60% in metastases vs. 48% in local disease; **Fig 1C)**. We believe the lower prevalence of non-metastatic ER+ samples in our cohort relative to traditional prevalence estimates is caused by treatment landscape and prognosis, which both impact the utilization of genomic profiling in standard clinical practice. The prevalence of HER2 amplification was similar in metastatic and local tumors (9.4% in metastases vs. 8.7% in local disease; **Fig 1B**). ER and HER2 status differed significantly across metastatic sites, with higher rates of ER positivity in liver and bone, lower rates in brain and lung, and with high prevalence of HER2 amplification in brain (p<1e-6 for association between subtype and site; **Fig 1D**) [44].

Histological subtype information was available for 50% of samples. Of these, 4,896 (84%) were categorized as invasive ductal carcinoma (IDC) and 611 (10%) as invasive lobular carcinoma (ILC) with the remainder from several rare subtypes including metaplastic carcinoma (n = 158), neuroendocrine carcinoma (n = 43), inflammatory carcinoma (n = 29), adenoid cystic carcinoma (n = 22), phyllodes tumors (n = 21), and mucinous carcinoma (n = 21). These constitute a substantial expansion of genomic profiling for rare breast cancers (**Fig 1E**). The genomic landscapes of these rare subtypes differ substantially from IDC and ILC, and can be found in **S2 Table**. Histological subtype was significantly associated with metastatic biopsy site, with ILC metastasizing at higher rates to the ovary and gastrointestinal tract (p<1e-6; **Fig 1F**), although the full extent of all metastatic sites within an individual cannot be determined from this data. For the 5,770 tumors without histological subtype, we inferred the probability that a tumor was IDC or ILC with 95% accuracy using a machine learning algorithm (**S1 Fig)**.

### Overview of genomic alterations

All samples underwent targeted sequencing using the FoundationOne® assay, which interrogates the coding sequences of cancer-associated genes as well as introns from genes frequently rearranged in solid tumors [33]. This assay provides a sensitive and specific readout of mutations, including oncogenic germline polymorphisms, copy number alterations, and structural rearrangements. Analyses were restricted to a set of 287 genes included on all versions of the assay (**S8 Table**). Because this targeted assay is used during standard clinical care we had access to a large patient population, though targeted sequencing does not provide the complete portrait created by whole exome or whole genome sequencing. The high *TP53* mutation rate relative to prior studies and the presence of *ESR1* mutations suggest that both the local and metastatic tumors in this dataset are enriched for patients with poor prognosis [14,45,46]. Samples had an average of 6.3 mutations (6.7 in metastases vs 5.6 in local disease, p<2e-16), 5.1 copy number alterations (5.3 in metastases vs 4.9 in local disease, p = 0.003), and 0.55 structural rearrangements (0.53 in metastases vs 0.56 in local disease, p = 0.07). One-hundred sixteen samples harbored mutations in more than 25 genes, with a significant enrichment of this hypermutated phenotype in metastatic samples relative to local samples (p<1e-5) and in ER+ samples (p<1e-08) [47]. A diverse set of genomic alterations were observed at high frequency across the 11,616 samples (**Fig 1G**), including mutations in *TP53* (55.9% of samples), *PIK3CA* (32.4%), *CDH1* (11%), *GATA3* (10.9%), *ESR1* (10.2%), and *KMT2D* (9.5%); amplifications of *MYC* (22.8%), *CCND1* (17.4%), and *HER2* (9.9%); deletions of *PTEN* (5.7%), *CDKN2A/B* (5%), and *RB1* (2.5%); and structural rearrangements of *HER2* (1.5%) and *FGFR1* (1.3%). *BRCA1/2* sequence variants (including deleterious mutations, variants of unknown significance, and deleterious germline variations) were also highly prevalent, at frequencies of 5.6% for *BRCA1* and 7.2% for *BRCA2*. These alterations have been consistently associated with breast cancer in prior reports [14,15,17,24,45,48]. Here we show that most of these frequently altered genes are enriched in a particular subtype based on ER/HER2 status (**S2 Fig, S1 Table**) or histology (**S2 Table**).

### Genomic alterations enriched in metastatic breast cancer

**Fig 2:**
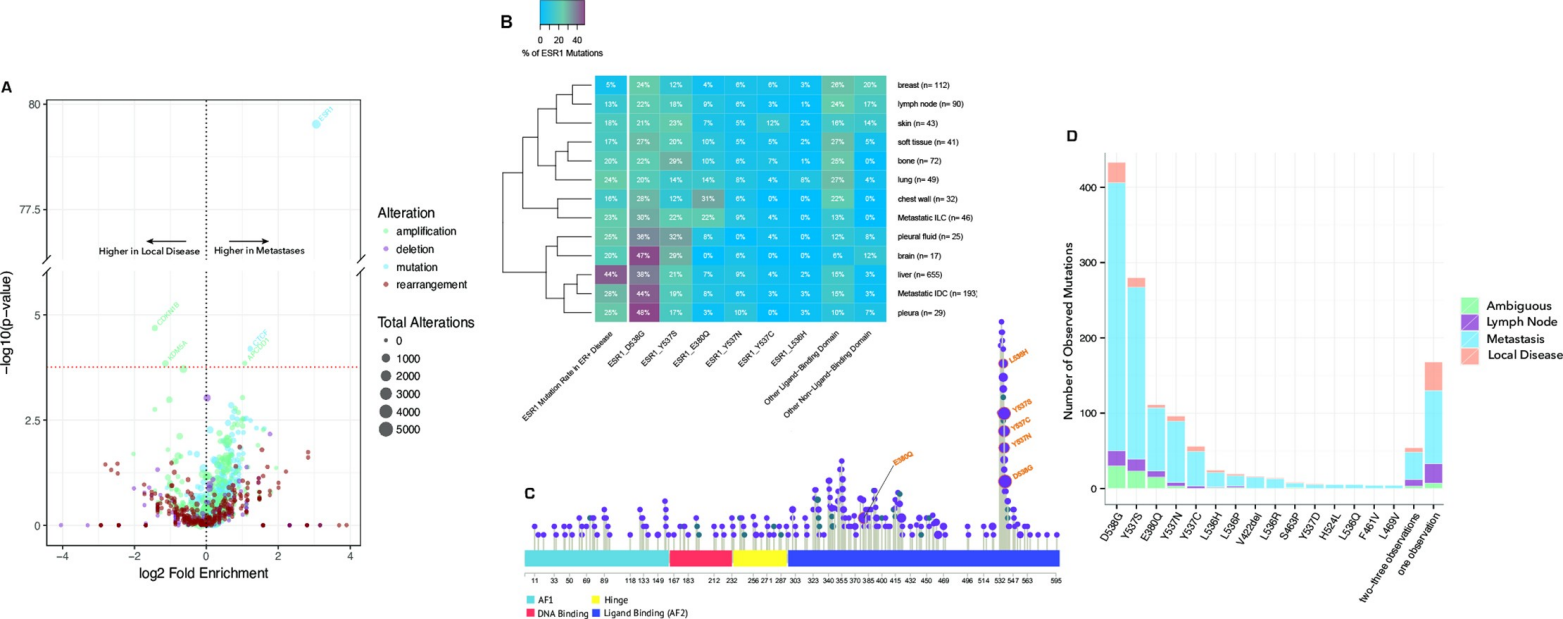
Comparison of metastatic tumors and local disease. (a) Enrichment analysis for alterations occurring at different rates in metastatic tumors vs. local disease, controlling for mutation load and molecular subtype (ER status and *HER2* amplification). (b) Prevalence of *ESR1* hotspot mutations by metastatic site and histological subtype. The far-left column represents the percent of patients with at least one *ESR1* mutation. All other columns represent the percentage of *ESR1* mutations of a certain type that we observe within a specific patient stratification (i.e., the top-left corner shows that 27% of the *ESR1* mutations we see in soft tissue samples are D538G). *ESR1* hotspots occur at significantly different frequencies at different metastatic sites, a result that we do not observe for other genes. (c) Distribution of *ESR1* mutations in the cohort, sized by prevalence. The majority of mutations occur within the ligand-binding domain. (d) Number of *ESR1* mutations by hotspot. All mutations observed 4 or more times are shown. Mutations observed 2–3 times, or 1 time, were pooled for analysis.

To search for alterations enriched in metastatic breast cancer we compared local and metastatic tumors while accounting for subtype (ER/HER2) and mutation load. Mutation load can explain the majority of the observed increase in mutation frequency for some genes, particularly large genes without clear hotspots. We confirmed the significant enrichment for *ESR1* mutations in metastatic tumors (18.3% in metastases vs. 2.2% in local disease, p<3e-80; **Fig 2A, S3 Table, S5–S7 Tables**). Beyond this principal feature of metastatic breast cancer, we found a previously unreported enrichment for *CTCF* mutations in metastatic samples (2% in metastases vs. 0.9% in local disease, p<2e-5; **Fig 2A**). Mutations in *CTCF* and at CTCF binding sites have previously been associated with multiple forms of cancer, putatively disrupting the epigenetic regulation of proliferation [49,50]. Furthermore, CTCF has been associated with epithelial-to-mesenchymal transition—a developmental process via which cells gain migratory and invasive properties that can be hijacked during cancer metastasis [51]. As such, we speculate that *CTCF* mutations are a metastatic driver in up to 2% of metastatic breast cancers.

In addition, we observed a significantly higher rate of *CDKN1B* (p27^kip1^) *amplification* in local tumors (1.3% in metastases vs. 3.6% in local disease, p<2e-5) (**Fig 2A**). Strengthening this result, the opposite alterations, *CDKN1B deletions* (0.2% in metastases vs. 0.1% in local disease, p = 0.09) and *mutations* (1.9% in metastases vs. 1.1% in local disease, p = 0.05), trend toward significant enrichment in metastatic tumors. *CDKN1B* controls cell cycle progression at G1 via inhibition of CDK4/6, and high expression of CDKN1B is a positive prognostic biomarker in early-stage disease [52]. A series of CDK4/6 inhibitors have recently emerged for the treatment of late-stage ER+ breast cancer patients that are particularly effective when used in conjunction with hormone therapy [53,54]. One possible interpretation of our data is that *CDKN1B* amplification in primary tumors acts to slow the rate of tumor proliferation and metastasis, which would point toward the possible utility of CDK4/6 inhibitors as a means of delaying progression to metastatic disease.

We also observed significantly higher rates of amplification of the FGFR ligands *FGF3*, *FGF4* and *FGF19* in metastases relative to local tumors, both for ER+ disease (27% in metastases vs. 17% in local disease, p = 0.004, 0.0007, 0.0015 for *FGF3*, *FGF4*, *FGF19*, resp.; **S6 Table**) and ER- disease (13% in metastases vs. 4% in local disease, p = 0.0008, 0.0001, 0.0002, resp.; **S7 Table**), when analyzing 1,405 samples with known ER status (*FGF* amplification did not reach significance in the full cohort because of a strong association with ER status). FGF signaling has been previously implicated in resistance to endocrine therapy [55]. Lastly, when only considering variants with known or likely tumorigenic potential, we found a significant enrichment of *KRAS* and *NF1* mutations in metastatic tumors (*KRAS*—1.9% in metastases vs. 1.1% in local disease, p = 0.0045; *NF1*–4.7% vs. 3.3%, p = 0.013, **S5 Table**), which suggests a potential role for the ras/MAPK pathway in metastasis [30].

### Site and subtype association of ESR1 hotspot mutations

Next we provided a comprehensive portrait of the prevalence and diversity of *ESR1* mutations in metastatic breast cancer. *ESR1* mutations are found in 1,183 tumors, 8.9% of those carry more than one *ESR1* mutation, and 922 (78%) are metastases. The *ESR1* mutation rate is highest in ER+ liver metastases (44%), followed by pleura (25%), lung (24%) and bone (20%) (**Fig 2B**). The mutation rate in ER+ brain metastases was 20%, contrary to the absence of *ESR1* mutations at this site in prior studies [56]. The most prevalent *ESR1* mutations are gain-of-function mutations in the ligand-binding domain, which have been shown to confer constitutive activity in the absence of estrogen (**Fig 2C**): D538G – 33.2% of all *ESR1* mutations; Y537S – 21.4%; E380Q – 8.5%; Y537N – 8.0%; Y537C – 4.3%; L536H –1.9%; and V422del –1.4%. We also see enrichment for rare *ESR1* mutations in metastases relative to local disease (p<1e-04 for the set of mutations seen twice; p<1e-05 for the set of mutations seen once) (**Fig 2D**). This effect is confined to the ligand-binding domain of *ESR1* (p = 0.004 for the set of mutations seen once in the ligand-binding domain vs. p = 0.9 for the set of mutations seen once outside the ligand-binding domain; p = 0.04 comparing enrichment between the two sets, demonstrating that the difference is not attributable to the larger number of mutations within the ligand-binding domain), suggesting that a long tail of mutations in the *ESR1* ligand-binding domain represents additional resistance mechanisms to aromatase inhibition.

The prevalence of these hotspot mutations further varies by site of metastasis (p<7e-7) and the histological subtype of the tumor (p = 0.003) (**Fig 2B**). In terms of metastatic site, visceral tissue (liver, pleura, pleural fluid, brain, and lung [57]) has significantly more D538G mutations (20–48% of ESR1 mutations) than all other *ESR1* hotspot mutations, including Y537S (p = 0.007). Bone metastases (non-visceral), on the other hand, have significantly more Y537S mutations (29%) relative to D538G (22%). Peripheral tissue (chest wall) has increased prevalence of E380Q mutations (31%), and both local breast tumors and lymph node metastases have higher rates of likely passenger mutations outside the ligand-binding domain. In terms of histological subtype, the *ESR1* hotspot mutations in invasive ductal carcinoma reflect visceral disease (44% are D538G). Those in invasive lobular carcinoma are enriched for E380Q mutations (22% are E380Q).

### Genomic alterations enriched at specific metastatic sites

**Fig 3:**
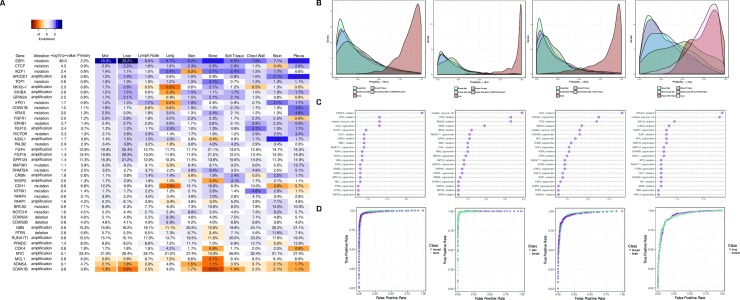
Mutations associated with specific metastatic sites. (a) Alterations enriched at specific metastatic sites. P-values represent comparison between all metastases and local disease. Each cell represents the rate of a specific alteration at a specific metastatic site, colored by enrichment or depletion relative to all local tumors. (b) Probability distributions for a panel of validation samples, using machine learning algorithms trained to differentiate breast tumors from bone, skin, brain, and lung tumors (left to right) using genomic features. Alterations enriched at each metastatic site were not included in the classifiers, but were used to stratify patients—to determine if any of the observed enrichments could be explained by misdiagnosis of new primary tumors. (c) Variable importance for the machine learning algorithms used in (b). The x-axis represents the mean decrease in accuracy of the classifier when a variable is permuted and indicates how useful a specific alteration is in determining the tissue of origin of a tumor from the set of genomic alterations it harbors. (d) ROC curves for the machine learning algorithms used in (b). Each point represents the true and false positive rate for one indication using one threshold on the model output to make a classification decision. A larger area under the curve represents a more accurate model, and we achieve high accuracy in all cases (93.8%, 96.6%, 91.7%, and 85.9% from left to right).

We next searched for site-specific metastatic alterations by comparing the genomic profiles of tumors from *specific* metastatic sites with local tumors while controlling for subtype and mutation load. We find multiple significant associations between genomic alterations and the site of metastasis (**Fig 3A, S4 Table**), the most intriguing of which include an enrichment of *ASXL1* amplifications (4.2% vs. 0.8% in local disease, p<2e-05) and *PTEN* deletions (11.8% vs. 5%, p<1e-04) in brain metastases; an enrichment of *DNMT3A* mutations in bone metastases (6.4% vs. 2.5%, p<2e-5); an enrichment of *NOTCH1* mutations in skin metastases (8.8% vs. 4.5%, p = 5e-4); and enrichments of *KRAS*, *KEAP1*, *STK11* and *EGFR* mutations in lung metastases (2.6–3.2% vs. 1.0–2.1%, p = 0.004–0.14). The latter enrichment strengthened when only considering known and likely driver mutations (2.8, 1.2, 2.4, 1.8% vs. 1, 0.3, 1, 0.6%, p = 0.0003, 0.003, 0.007, 0.004, respectively).

Most of these enriched alterations have been associated with primary tumors at the site of metastasis. *KRAS* is the most common driver mutation in lung cancer, and is highly co-occurrent with *KEAP1* and *STK11* mutations [58,59], *NOTCH1* has a putative role in skin cancer [60], loss of *PTEN* is the most common genomic alteration in glioblastoma [61], and *DNMT3A* has been associated with leukemia and myelodysplasia [62,63]. This suggests that these alterations serve to mimic primary tumors at the site of metastasis and confer adaptation to the local tumor microenvironment. To further support this hypothesis, we ensured that these tumors are not misdiagnosed *de novo* primary tumors occurring in patients with a prior history of breast cancer. For this purpose, we developed machine learning algorithms to differentiate breast tumors from primary tumors of the brain, bone, lung, and skin (**Fig 3B, 3C and 3D**). The algorithms were trained using genomic data from 500 tumors of each indication, sequenced during standard care using the FoundationOne assay, with the enriched alterations associated with each indication masked (e.g., *PTEN* deletions excluded from the brain classification). In each case, we achieved high accuracy on held-out test data for the prediction of skin vs. breast (96.6%), lung vs. breast (85.9%), bone vs. breast (93.8%), and brain vs. breast (91.7%) (**Fig 3D**). We then applied the algorithms to the metastatic breast tumors containing the enriched alterations, to confirm that those tumors are equally likely to originate from breast tissue relative to all other metastatic breast tumors in the dataset. Fourteen percent of breast tumors with *DNMT3A* mutations that metastasized to bone were classified as bone, statistically indistinguishable from the 12% of all breast metastases classified as bone (p = 0.39). Similarly, 7% of brain metastases that harbor *ASXL1* amplifications or *PTEN* deletions were classified as brain, less than the 9% of all breast metastases classified as brain. Skin metastases that harbor *NOTCH1* mutations were classified as skin cancer at a higher rate (18% vs 5%, p = 0.052), but the effect was primarily driven by differences in subtype prevalence between local tumors and skin metastases and was not significant after controlling for this (p = 0.78). These results are consistent with the hypothesis that the enrichments for *DNMT3A* mutations in bone metastases, *NOTCH1* mutations in skin metastases, and *ASXL1* amplifications/*PTEN* deletions in brain metastases are biologically relevant, either as adaptations that arise in response to the local tumor microenvironment or as drivers of site-specific patterns of metastasis. An alternative hypothesis for the enrichment of *DNMT3A* mutations is clonal hematopoiesis of unknown potential, a process in which somatic mutations in hematopoietic stem cells lead to the outgrowth of distinct subclones that have been associated with cancer [64–66]; we provide evidence for this hypothesis in S3 Fig.

We observed a different pattern in breast tumors that metastasize to lung and harbor a mutation in *KEAP1*, *KRAS*, *STK11*, or *EGFR* (**Fig 3B**). The lung classifier, which does not consider mutations in these four genes, scored a significantly larger fraction of these tumors as lung compared to all breast metastases (50% vs. 18%, p = 2e-5, n = 52). Furthermore, we observed that breast cancer metastases at *any* site that harbor mutations in *KEAP1*, *KRAS*, and *STK11*, a triplet commonly associated with lung cancer, genomically resemble lung tumors (100% vs. 18%, p = 2e-8, n = 13). Consistent with these findings, we found enrichment for additional lung-associated alterations in this set of tumors including mutations in *LRP1B* (21.2% vs. 8.2%, p = 0.03) and deletions of *CDKN2A/B* (11.5% vs. 4.5%, p = 0.02). These findings are highly suggestive of misdiagnosis (primary lung tumors diagnosed as breast metastases and lung cancer metastases diagnosed as breast cancer metastases). Misdiagnosis has substantial implications for treatment choice and efficacy and illustrates the potential of genomic profiling to complement other modalities in selecting effective treatments for individual patients.

## Discussion

Large-scale sequencing of cancer genomes holds the promise of delivering novel therapeutic targets and personalized treatment for patients. Initial efforts to characterize and understand the cancer genome focused on primary disease, and led to the development of multiple targeted therapies with substantive impact on patients’ lives. We have conducted a comprehensive analysis of real-world breast cancer metastases sequenced during standard clinical care and show enrichment for (i) mechanisms of acquired resistance and (ii) alterations that may induce or accelerate metastasis. It is our hope that a deeper understanding of these classes of alterations will ultimately lead to new treatments for metastatic disease, which is the cause of most deaths in breast cancer and represents a substantial unmet medical need.

Mutations in the *ESR1* ligand-binding domain are the principal feature of hormone receptor-positive metastatic breast cancer, arising in response to aromatase inhibition and allowing the tumor to progress in the absence of estrogen [67,68]. Therapies that modulate the mutant receptor are in clinical trials, and *ESR1* mutations in circulating tumor DNA are a promising biomarker for disease progression [26,69]. We have shown that specific *ESR1* hotspot mutations are associated with specific metastatic niches and disease histologies, suggesting the possibility of cofactor interactions or biological contexts that could be druggable; prior work has demonstrated functional differences between ESR1 hotspot mutations *in vitro* and shown that hotspots differentially respond to drug [56,70]. The development of targeted therapy against ESR1 mutations is an active area of research with great promise for treating metastatic disease, and a deeper understanding of how specific mutations function *in vivo* will be crucial for optimizing patient outcomes. In addition, we provide evidence for a long tail of ligand-binding domain mutations that appear to be functional given their enrichment in metastatic disease. Patients with these rare mutations may be resistant to traditional hormone therapy and should be monitored closely for disease progression.

The enrichment for *CTCF* mutations that we see in metastatic disease is not an anticipated resistance mechanism. *CTCF* binding site mutations are enriched in multiple cancers, and *CTCF* mutations in cancer have been shown to specifically alter interactions with promoters or insulators of genes associated with proliferation [50,51]. The observed enrichment is consistent with the hypothesis that multiple steps in the metastatic cascade involve epigenetic transitions that allow tumor cells to co-opt biological processes to promote growth and metastasis.

In order to colonize a distal site, tumor cells need to disseminate, evade the immune system, and adapt to a novel microenvironment. It is an open question whether this metastatic potential is present in primary disease or is enabled by additional genetic and epigenetic events. We have shown multiple instances in which breast cancer metastases are enriched for alterations that occur in primary tumors at the site of metastasis. This is consistent with the hypothesis that, in some contexts, metastases mimic primary tumor biology to adapt to novel microenvironments, but experimental follow-up will be necessary to fully understand this process.

When a patient with a history of cancer presents with a malignancy at a new site, the decision whether the lesion is a metastasis or a novel primary tumor has a dramatic impact on prognosis and treatment. Diagnosis is complicated by the possibility that the metastasis has arisen from a subclone of the primary tumor. Microsatellite analysis has been used for differential diagnosis, and traditional methods include histology and interval to cancer formation [71]. We have shown the potential of an orthogonal approach, leveraging machine learning and large-scale sequencing data to classify the most probable tissue of origin of a metastatic lesion. Using this method, we found multiple instances of likely primary lung tumors misdiagnosed as breast metastases, though we cannot exclude the possibility that these represent breast tumors that strongly mimic lung cancer biology.

## Conclusions

Besides the known hotspot mutations in *ESR1*, we observed a metastatic enrichment of previously unreported, lower-prevalence mutations in the ligand-binding domain, implying that these mutations may also be functional. Furthermore, individual *ESR1* hotspots are significantly enriched in specific metastatic tissues and histologies, suggesting functional differences between these mutations. Other alterations enriched across all metastases include loss of function of the CDK4 regulator *CDKN1B*, and mutations in the transcription factor *CTCF*. Mutations enriched at specific metastatic sites generally reflect biology of the target tissue and may be adaptations to growth in the local environment. These include *PTEN* and *ASXL1* alterations in brain metastases and *NOTCH1 alterations* in skin. We observed an enrichment of *KRAS*, *KEAP1*, *STK11* and *EGFR* mutations in lung metastases. However, the patterns of other mutations in these tumors indicate that these are misdiagnosed lung primaries rather than breast metastases.

A core implication of this paper, as well as other recent work on cancer metastasis in different indications and utilizing different assays, is that there are perhaps fewer specific genomic alterations that drive metastasis than was anticipated [28,29]. This suggests several non-exclusive possibilities. First, the majority of primary tumors may harbor metastatic potential without the need to incur additional genomic alterations. Second, changes in cell state during the process of metastasis may be primarily epigenetic rather than genetic in nature. Third, metastasis may be driven by a large number of alterations with small individual effects.

Cancer is a heterogeneous disease—each patient presents with a unique constellation of genetic and epigenetic alterations that have transformed healthy tissue into a malignancy. We are in the early stages of embracing and understanding this complexity, but doing so will allow us to develop new therapies and target the right patients with the right drugs. This work, which utilizes real-world data that lacks extensive clinical annotation but provides enormous scope and scale, is complementary to efforts that generate curated data in small cohorts. Each has a comparative advantage in tackling specific questions, but both may be necessary to realize the promise of genomic medicine, deliver effective personalized oncology, and ultimately improve outcomes for patients.

## Supporting information

S1 Fig Machine learning algorithms to classify molecular and histological subtype. (a) Probability distributions for the output of machine learning algorithms trained to infer molecular (left) or histological (right) subtype from the set of genomic alterations harbored by a tumor. See Fig 3B legend for more details. (b) Variable importance for the machine learning algorithms used in (a). The x-axis represents the mean decrease in accuracy of the classifier when a variable is permuted and indicates how useful a specific alteration is in determining the subtype of a tumor from the set of genomic alterations it harbors. (c) ROC curves for the machine learning algorithms used in (a). See Fig 3B–3D legend for more details.
(TIF)

S2 Fig Landscape of genomic alterations by molecular subtype. Landscape of genomic alterations in (a) ER+, (b) ER-, and (c) HER2+ disease. Each cell represents the status of one gene in one patient, colored by alteration type. ER status was determined by pathology report. HER2 status was determined by *HER2* copy number.
(TIF)

S3 Fig Evidence for clonal hematopoiesis. Clonal hematopoiesis is a process via which somatic mutations in hematopoietic stem cells lead to the outgrowth of distinct subclones [64]. Clonal hematopoiesis is observed in 10% of adults over 65 years of age, but in only 1% of those under 50, and has been associated with cancer [65,72]. *DNMT3A* mutations are the most frequently observed mutation in clonal hematopoiesis of indeterminate potential (CHIP) [64], and have not previously been associated with breast cancer. As such, we speculated that the observed enrichment of *DNMT3A* mutations in bone metastases might be a consequence of clonal hematopoiesis and not of alterations harbored by the tumor. Consistent with this hypothesis, we observe an increasing mutation rate with patient age (a) that cannot be explained by changes in histological and molecular subtype (c) and a decreasing fraction of reads associated with the mutant allele that we do not observe in other genes (b). The enrichment is not specific to bone metastases, but the rate at which clonal hematopoiesis may be present varies by biopsy site (d). (a) Frequency of mutation by patient age, normalized to the observed frequency in patients aged 20–39, for genes that show the strongest association with patient age. Most effects can be explained by changing proportions of histological and molecular subtype, seen in Fig 1D and 1F. *DNMT3A* mutations increase with age and show a unique pattern. (b) Fraction of reads associated with the mutant allele in patients that harbor a mutation for *PIK3CA*, *TP53*, and *DNMT3A*. The read fraction for *DNMT3A* decreases with patient age, consistent with CHIP. (c) Prevalence of histological and molecular subtype by patient age. (d) *DNMT3A* mutation rate by patient age and biopsy site.
(TIF)

S1 Table Top alterations by molecular subtype, as defined by *HER2* copy number and ER status from pathology report, in 1,405 samples with complete clinical annotation. Pathology reports were scored by an algorithm with 95% accuracy.
(XLSX)

S2 Table Top alterations by histological subtype in male patients and patients under 40. (XLSX)

S3 Table Alterations enriched in metastatic tumors relative to local disease (primary tumors and local recurrences). Corrected p-values were calculated by permuting the met/local status of samples 1000 times, reflecting the probability of observing a more significant enrichment by chance.
(XLSX)

S4 Table Alterations enriched by site of metastasis relative to local disease (primary tumors and local recurrences). Corrected p-values were calculated by permuting the tissue of samples 1000 times. Results for the 9 most common biopsy sites are shown, for alterations that occurred at least ten times at the metastatic site.
(XLSX)

S5 Table Mutations enriched in metastatic tumors relative to local disease (primary tumors and local recurrences) after filtering out variants of unknown significance. Corrected p-values were calculated by permuting the met/local status of samples 1000 times, reflecting the probability of observing a more significant enrichment by chance.
(XLSX)

S6 Table Mutations enriched in ER+ metastatic tumors relative to ER+ local disease (primary tumors and local recurrences) as defined by IHC for samples with available IHC (n = 719). Corrected p-values were calculated by permuting the met/local status of samples 1000 times, reflecting the probability of observing a more significant enrichment by chance.
(XLSX)

S7 Table Mutations enriched in ER- metastatic tumors relative to ER- local disease (primary tumors and local recurrences) as defined by IHC for samples with available IHC (n = 532). Corrected p-values were calculated by permuting the met/local status of samples 1000 times, reflecting the probability of observing a more significant enrichment by chance.
(XLSX)

S8 Table. Genes included on FoundationOne Panels (XLSX)

## List of abbreviations

AKT1: AKT serine/threonine kinse 1
ASXL1: ASXL transcriptional regulator 1
BRCA1/2: BRCA 1/2 DNA repair associated
CBFB: core-binding factor subunit beta
CTFC: transcriptional repressor CTFC
CCND1: cyclin D1
CDH1: cadherin 1
CDK4: cyclin-dependent kinase 4
CDKN1B: cyclin dependent kinase inhibitor 1b
CDKN2A/B: cyclin dependent kinase inhibitor 2a/b
CTCF: CCCTC-binding factor
DNA: deoxyribonucleic acid
DNMT3A: DNA methyltransferase 3 alpha
EGFR: epidermal growth factor receptor
ERBB2: erb-b2 receptor tyrosine kinase 2
ESR1: estrogen receptor 1
FFPE: formalin-fixed paraffin-embedded
FGFR: fibroblast growth factor receptor
GATA3: GATA binding protein 3
H&E: hematoxylin and eosin stain
HER2: erb-b2 receptor tyrosine kinase 2
KEAP1: kelch like ECH associated protein 1
JAK/STAT: Janus kinase/signal transduce and activator of transcription
KMT2D: lysine methyltransferase 2D
KRAS: KRAS proto-oncogene
MAF: minor allele frequency
MAPK: mitogen activated kinase-like protein
MAP3K1: mitogen-activated protein kinase kinase kinase 1
OCR: optical character recognition
PIK3CA: phosphatidylinositol-4,5-biphosphate 3-kinase catalytic subunit alpha
PTEN: phosphatase and tensin homolog
RB1: transcriptional corepressor 1
SERM: selective estrogen receptor modulator
SERD: selective estrogen receptor degrader
STK11: serine/threonine kinase 11
SWI/SNF: Switch/Sucrose non-fermentable
TP53: tumor protein p53
