# Supplementary figures and images for: The genomic landscape of metastatic breast cancer: Insights from 11,000 tumors
Source: PLoS One. 2020 May 6;15(5):e0231999. doi: 10.1371/journal.pone.0231999 (PMC7202592; doi:10.1371/journal.pone.0231999)

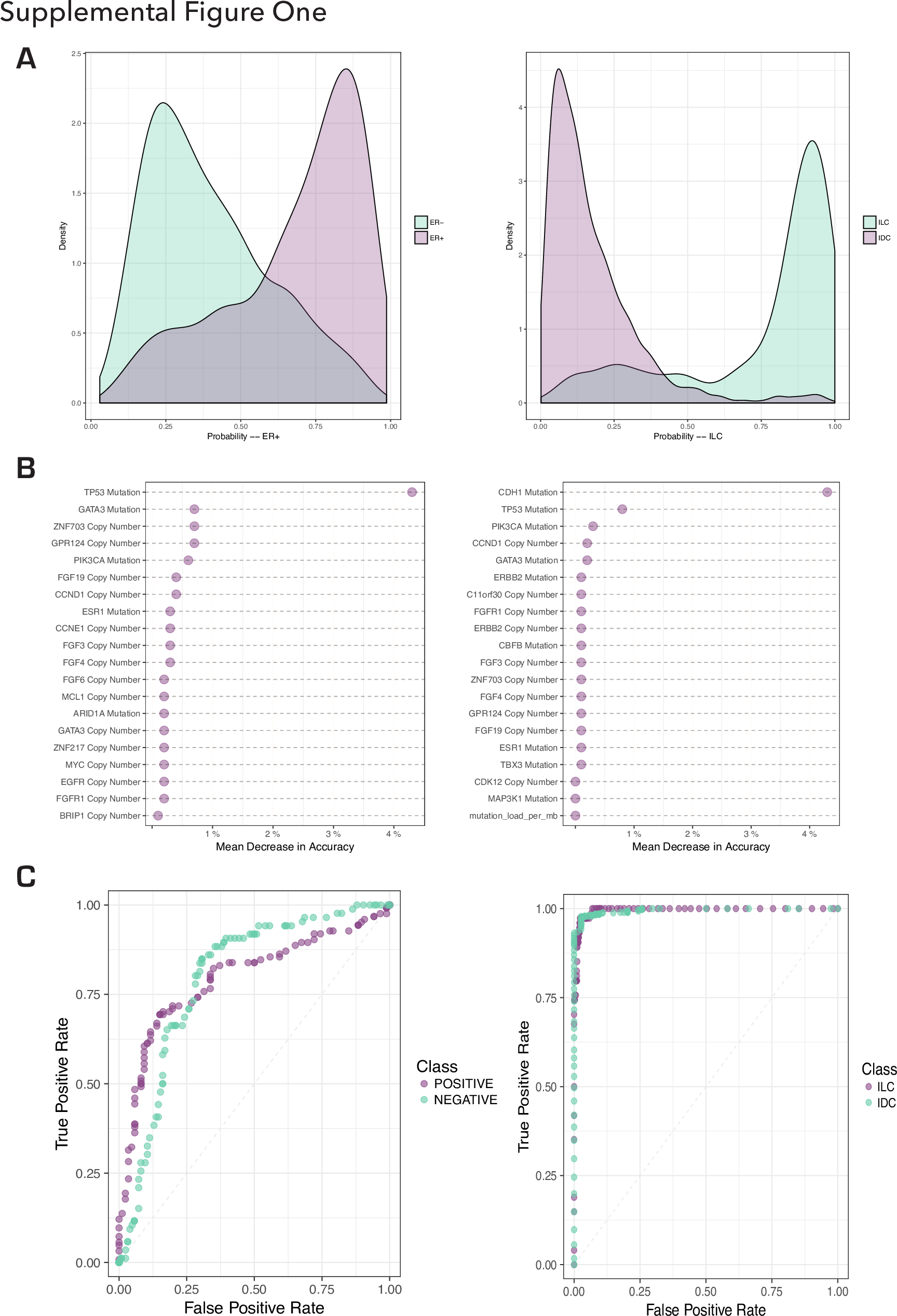

Supplement: S1 Fig — (a) Probability distributions for the output of machine learning algorithms trained to infer molecular (left) or histological (right) subtype from the set of genomic alterations harbored by a tumor. See Fig 3B legend for more details. (b) Variable importance for the machine learning algorithms used in (a). The x-axis represents the mean decrease in accuracy of the classifier when a variable is permuted and indicates how useful a specific alteration is in determining the subtype of a tumor from the set of genomic alterations it harbors. (c) ROC curves for the machine learning algorithms used in (a). See Fig 3B–3D legend for more details. (TIF) [file pone.0231999.s001.tif]

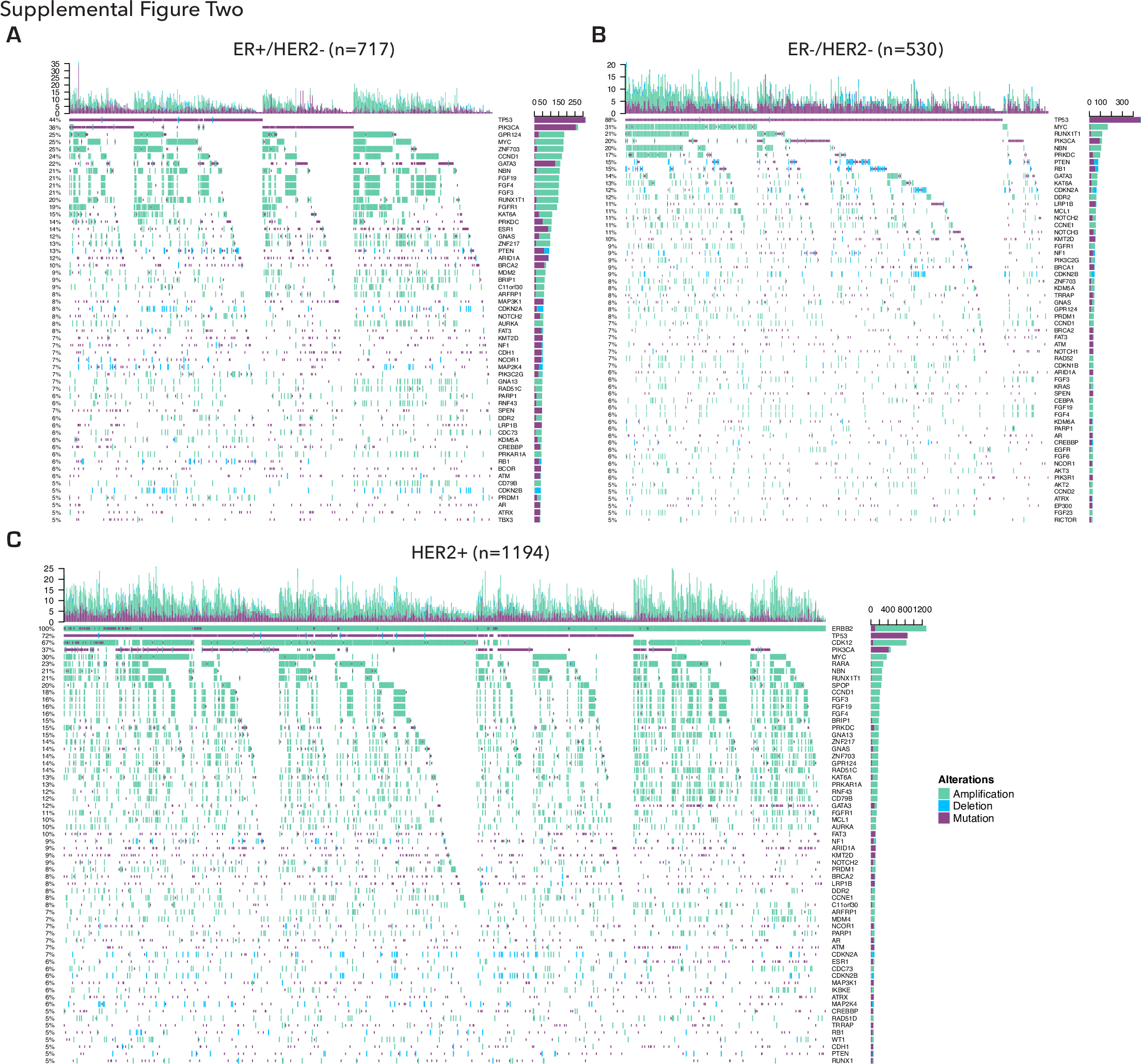

Supplement: S2 Fig — Landscape of genomic alterations in (a) ER+, (b) ER-, and (c) HER2+ disease. Each cell represents the status of one gene in one patient, colored by alteration type. ER status was determined by pathology report. HER2 status was determined by HER2 copy number. (TIF) [file pone.0231999.s002.tif]

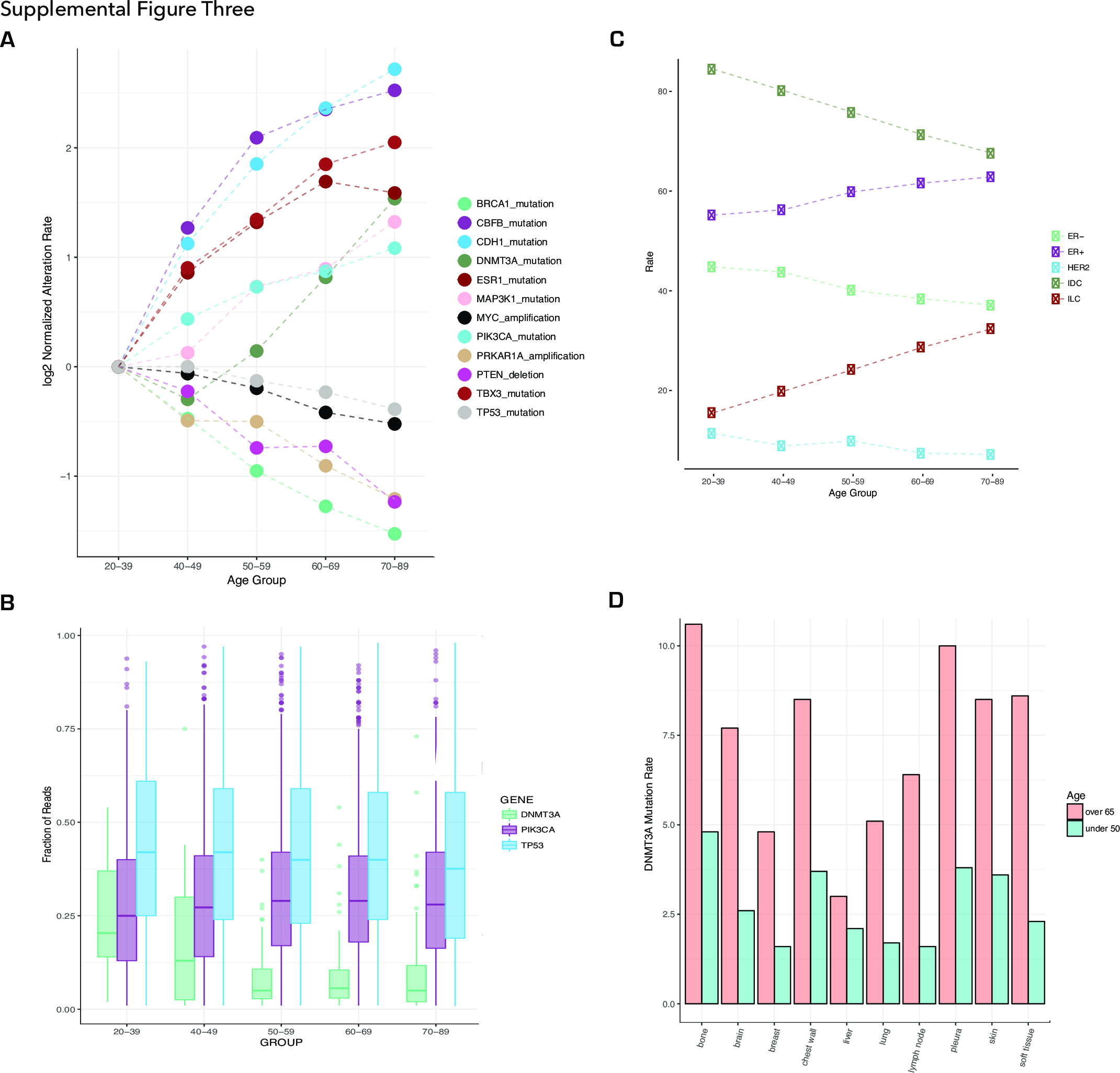

Supplement: S3 Fig — Clonal hematopoiesis is a process via which somatic mutations in hematopoietic stem cells lead to the outgrowth of distinct subclones [64]. Clonal hematopoiesis is observed in 10% of adults over 65 years of age, but in only 1% of those under 50, and has been associated with cancer [65,72]. DNMT3A mutations are the most frequently observed mutation in clonal hematopoiesis of indeterminate potential (CHIP) [64], and have not previously been associated with breast cancer. As such, we speculated that the observed enrichment of DNMT3A mutations in bone metastases might be a consequence of clonal hematopoiesis and not of alterations harbored by the tumor. Consistent with this hypothesis, we observe an increasing mutation rate with patient age (a) that cannot be explained by changes in histological and molecular subtype (c) and a decreasing fraction of reads associated with the mutant allele that we do not observe in other genes (b). The enrichment is not specific to bone metastases, but the rate at which clonal hematopoiesis may be present varies by biopsy site (d). (a) Frequency of mutation by patient age, normalized to the observed frequency in patients aged 20–39, for genes that show the strongest association with patient age. Most effects can be explained by changing proportions of histological and molecular subtype, seen in Fig 1D and 1F. DNMT3A mutations increase with age and show a unique pattern. (b) Fraction of reads associated with the mutant allele in patients that harbor a mutation for PIK3CA, TP53, and DNMT3A. The read fraction for DNMT3A decreases with patient age, consistent with CHIP. (c) Prevalence of histological and molecular subtype by patient age. (d) DNMT3A mutation rate by patient age and biopsy site. (TIF) [file pone.0231999.s003.tif]
